# Evaluation of Local Food Baits for Monitoring Fruit Flies (Diptera: Tephritidae) in Guava Orchards in Maputo, Mozambique

**DOI:** 10.3390/insects17060555

**Published:** 2026-05-28

**Authors:** Deborah Apio, Laura Canhanga, Domingos Cugala

**Affiliations:** 1 Department of Agronomy and Forestry Engineering, Eduardo Mondlane University, Avenida Julius Nyerere, n° 3453, Maputo P.O. Box 257, Mozambique; laura.canhanga@uem.ac.mz (L.C.); dcugala@gmail.com (D.C.); 2 Centre of Excellence in Agri-Food Systems and Nutrition (CE-AFSN), Eduardo Mondlane University, Praça 25 de Junho Edificio da Reitoria 5° Andar, Maputo P.O. Box 257, Mozambique; 3 Department of Agriculture, Uganda National Institute for Teacher Education, Kaliro Campus, Plot No 21, Block 15, Buwenge Road, Kaliro District, Kaliro P.O. Box 65, Uganda

**Keywords:** Tephritidae, palm sap, molasses, torula

## Abstract

Fruit flies (Diptera: Tephritidae) are major pests of crops that require effective monitoring for management. This study evaluated locally available food baits for monitoring fruit flies in guava orchards in Maputo, Mozambique. It also assessed infestation levels and examined the relationship between trap catches and the ratio adult/kg of fruit. Four treatments (palm sap, molasses, torula yeast, and water) and four replications were used. Tephri traps were installed on four trees per block and inspected weekly, while guava fruits from both trees and the ground were collected and incubated to estimate infestation indices. Three genera (*Bactrocera*, *Dacus*, and *Ceratitis*) were recorded, with a high relative abundance (90.37%) for *Bactrocera dorsalis.* Torula and palm sap were the best attractants, with no significant difference between them. Guava fruits showed high infestation (208.5 ± 13.3 adults/kg). Trap catches of *B. dorsalis* were positively correlated with adults/kg, suggesting 42.5% of infestation variation and highlighting the influence of factors outside the orchard on infestation indices. The results show that palm sap is a promising low-cost alternative bait and highlight the importance of considering area-wide management of fruit flies.

## Simple Summary

Fruit flies are serious pests that damage fruit and vegetables, reducing farmers’ income in many tropical regions. Effective monitoring is essential for managing these pests, but many existing tools are costly and not easily accessible to small-scale farmers. This study evaluated whether locally available food baits can be used to monitor fruit flies in guava orchards in Maputo, Mozambique. The study also provided systematic information on fruit fly infestation indices on guava and proposes a formula for predicting the level of infestation inside guava fruits based on the number of flies caught in traps. The results showed that palm sap (from *Phoenix reclinata*) was as effective as the commercial torula yeast bait in attracting fruit flies. The number of adult fruit flies/kg of fruit was high when the trap catches were high and vice versa. These findings show that palm sap is a reliable low-cost option for fruit fly monitoring and can support more effective and affordable pest management for farmers. Trap catches can be used to predict but may not fully explain infestation indices in orchards.

## 1. Introduction

Global fruit production exceeded 887 million tonnes in 2020. Guava (*Psidium guajava*), sometimes classified within the mango and mangosteen group, accounted for approximately 59.2 million tonnes, with the majority produced in India (about 44%), followed by Indonesia and China [1]. In Africa, guava is widely cultivated in subsistence and small-scale commercial systems; however, continent-wide production data remain fragmented [2]. In Mozambique, guava production, alongside mango (*Mangifera indica* L.) and mangosteen (*Garcinia mangostana*), reached 28.9 thousand tonnes in 2022 [3].

Guava, together with other fruit, plays an important role in human diet due to their nutritional value, contributing as an excellent source of vitamins and minerals essential in the regulation of almost all vital functions of the body [4]. Guava is valued not only for its fresh consumption but also for making juice, jams, and pulp, and for its healthy content of vitamins, fiber, and antioxidants [2]. However, the production of these fruits is put under a threat by infestation with fruit flies.

Fruit flies (Diptera: Tephritidae) are recognized globally as devastating pests of horticultural crops, particularly in tropical and subtropical regions where they can thrive year-round [5]. For example, fruit flies are estimated to cause an economic loss of more than US$2 billion across Africa annually [6]. In Mozambique, the invasive species *Bactrocera dorsalis* has become the most dominant and economically significant, causing infestation levels exceeding 90% in guava fruits [7,8]. This leads to huge direct losses by decreasing the quantity and quality of yields and indirect losses through the limitation of export trade [9,10,11].

Although chemical attractants and protein baits have been developed for the monitoring and management of fruit flies, they are often unaffordable and therefore may remain inaccessible to most small-scale farmers [12]. Food-based attractants are widely used because they provide protein and energy required by adult fruit flies, especially females during egg development. Among these attractants, protein hydrolysates and torula yeast are commonly used due to their ability to release ammonia and other volatile compounds that attract flies [13,14]. Fermentation plays a key role in the effectiveness of these attractants, since during this process microorganisms break down sugars and proteins, producing volatile compounds such as ethanol, acetic acid, and esters. These compounds are commonly associated with ripening or decaying organic matter and may act as cues for fruit flies when locating food and oviposition sites [15]. However, the performance of fermentation-based attractants depends on factors such as bait composition, preparation time, stage of fermentation, temperature, and humidity in the environment [16]. These factors influence the type and amount of volatiles released and therefore affect trap efficiency. Prior research has shown the efficacy of protein baits for trapping and controlling fruit flies, but has little been studied to assess the efficacy of baits that are locally available, like palm sap and molasses, especially in Mozambique. Also, information on detection sensitivity of these baits under low population densities of fruit flies remains scarce, and predicting adults’ infestation indices based on trap catches remains a challenge.

This research is aimed at assessing the efficacy of local food baits in attracting fruits flies and to estimate the infestation indices in guavas and the extent to which the number of trapped flies can reliably predict the number of adults/kg of fruit. The outcome will give farmers effective and cheaper means of monitoring than the conventional attractants, which is among the principal pillars of sustainable production of fruit and improvement of lives. Also, by estimating the correlation between the trapped flies and the adult flies that emerge from the incubated fruits, more effective management strategies can be recommended since they it provide information on the main source of fruit flies.

## 2. Materials and Methods

### 2.1. Description of the Study Area

The study was carried out in guava orchards (Lat. −26.0867, Long. 32.38998) in Umbeluzi Agrarian Station, Boane District, Maputo Province, Mozambique, starting from 21 March 2025 to 12 September 2025. It was conducted under typical tropical climatic conditions characterized by moderate temperatures, low to moderate rainfall, and relatively high humidity. Minimum temperatures ranged from 12.3 to 23.0 °C, while maximum temperatures ranged from 24.2 to 30.4 °C, with mean weekly temperatures varying between 17.9 and 26.3 °C. Rainfall during the study period was generally low, ranging from 0 to 4.07 mm per week, with several weeks recording no rainfall. Relative humidity remained relatively high throughout the study, ranging from 56.7% to 80.5%. These conditions are known to influence fruit fly activity and attractant performance [16]. The site was selected based on the dominance of guava trees, which are some of the preferred hosts by fruit flies [7], and since they were at the fruiting stage, they were expected to be hosting fruit flies. Other fruit within the vicinity of the orchard included mangoes, tropical almond (*Terminalia catappa*), oranges (*Citrus sinensis*), and bananas (*Musa* spp.), which are also important hosts of fruit flies.

### 2.2. Sampling Procedures

#### 2.2.1. Description of Treatments

The experiment was conducted in a randomized complete block design (RCBD) with 4 treatments and 4 replications (blocks). The treatments consisted of two test baits (palm sap and molasses) and two controls: torula yeast (positive control) and water (negative control). Within the guava orchards, four blocks (blocks 1–4) were selected and separated at least about 50 m from each other. At each block, 4 guava trees were selected randomly and marked as trees 1–4, separated at least 20 m apart from each other, containing the traps for the treatments (one trap per treatment per tree) [14,17,18].

For each treatment, Tephri traps from Insect Science (Pty) Ltd., Tzaneen, South Africa, containing 250 mL of the attractive solution per trap/week were used [19,20,21] as follows:

- Molasses, a by-product of refined sugar rich in fermentable sugars, was prepared by diluting 100 mL of molasses with 900 mL of clean water to make a 10% solution of molasses according to Alves et al. [22]. The solution was prepared fresh and weekly prior to field deployment to ensure consistency in fermentation and attractiveness. The raw material was obtained from a sugar factory located in Xinavane (Açucareira de Xinavane) (Manhiça district, Maputo, Mozambique) in bottles of 20 L.
- Palm sap was obtained from the inflorescence of the palm tree (*Phoenix reclinata*) [15]. It is a sugar-rich liquid containing sucrose, glucose, and fructose, which undergo rapid natural fermentation due to microbial activity [15]. Fresh palm sap was used and it was chosen because it is readily available in Mozambique. Fresh material was readily available from local vendors at Incoluane, Gaza Province, Mozambique. It was stored in 1 L plastic bottles under frozen conditions to preserve freshness. Prior to field use, the samples were transferred to a refrigerator and allowed to thaw for approximately 24 h before deployment in traps to maintain a similar fermentation stage across sampling periods.
- An aqueous solution of torula yeast (*Candida utilis*) was prepared by suspending one pellet (5 g) of torula yeast (Scentry Biologicals Inc., Billings, MT, USA) in 100 mL of clean water [14,23]. The torula yeast solution was prepared immediately before field placement.
- For water, 250 mL of clean tap water was placed in the Tephri traps and used as a negative control.

#### 2.2.2. Trap Placement and Monitoring

At each block, four guava trees were selected. As cited above, each tree received one trap containing a single treatment. The four treatments were randomly assigned so that each set of treatments represented one block. The traps were set up within the tree canopy, approximately 1.5 to 2 m above the ground surface and preferably in shaded locations [17,24]. Trap holes or entrances were left free of leaves and tree branches to allow easy access for fruit flies [18,25]. The trap-holding string was impregnated with vaseline to prevent the entry of ants and their subsequent predation on the attracted fruit flies. All traps were inspected once a week. The captured insects were removed and separated from the liquid attractant using a sieve, the suspension was discarded, and the captured insects were rinsed with water, placed in plastic vials containing 70% alcohol [24,26], and duly labeled according to the block, attractant, date, and week of collection. After collecting the samples, the traps were washed with water, the attractants were replaced with the fresh ones, and the position of the traps was changed clockwise to avoid potential position bias [10,12]. Used attractants were disposed of by pouring them away at a distant location to prevent re-attracting fruit flies to non-trap areas, which could reduce trap effectiveness and create false monitoring results [27]. Subsequently, all collected insects were taken to the laboratory of Entomology at the Faculty of Agronomy and Forestry Engineering (FAEF) in Maputo. In the laboratory, all fruit flies were separated from other captured individuals based on their general characteristics (wing venations). All fruit fly specimens collected were washed, identified, counted, and recorded.

### 2.3. Guava Fruit Sampling

Fruits were sampled every week from the ground and from trees in the four experimental blocks to estimate the infestation indices of fruit flies in the orchard. The number of fruits collected per block varied slightly across sampling weeks due to seasonal changes in fruit availability. On average, approximately 15–20 fruits per block were collected per week, with a maximum of 20 fruits (10 from the ground and 10 from the tree) when fruit availability was high. Fruits were selected randomly, regardless of visible infestation symptoms, to avoid sampling bias. Collected fruits were put into labeled containers and transported to the FAEF Entomology Laboratory for incubation. The fruits were counted, weighed, and incubated in groups of at least five fruits in mesh-top plastic containers lined with moist sterilized sand under laboratory conditions of temperature and humidity. Fruits collected from the ground were incubated separately from the ones collected from the trees. The containers were inspected twice every week to harvest any pupae [10,28]. The pupae were freed from the soil with a sieve, counted, and placed in plastic vials lined with moist paper for humidity. The larvae which had not yet developed into pupae were placed back in the plastic container with soil for pupation. The harvesting of pupae went on until two consecutive samplings yielded no pupae Then, the fruits were dissected to check for any larvae or pupae that might have failed to emerge [19]. The plastic vials containing the pupae were covered with perforated cloth for aeration and kept at room temperature until the adults emerged or for two weeks. The adults that emerged from the fruits, together with the fruit fly specimens trapped from the field were counted, separated by genera, and sexed before they were identified. The identification was based on the morphological features described by Ekesi & Billah and De Meyer et al. [9,19] with the aid of a magnifying glass and compared with specimens already identified. Also, electronic identification keys were used as described by Virgilio et al. [29]. For more precise identification, the specimens were submitted to fruit fly taxonomic specialists at the Royal Museum for Central Africa, Brussels, Belgium. The identified fruit flies from each species were sexed and counted.

### 2.4. Determination of Variables

#### 2.4.1. Estimation of the Absolute and Relative Abundance of Fruit Fly Species

The absolute abundance of fruit fly species captured in each treatment was estimated based on the total number of individuals of each species attracted in each treatment. This variable has been used in various studies to determine population size and dynamics of fruit flies in mango orchards [10,12]. Relative abundance was estimated as the percentage ratio between the number of individuals of each species found in each type of treatment and the total number of individuals of all species found for the same treatment. (Formula (1)) This approach has been similarly employed in the evaluation of fruit fly monitoring techniques and the efficacy of different attractants in diverse mango-growing regions [12,30].

(1) $$\mathrm{Ab}=\frac{n}{N}\times 100,$$

where Ab = relative abundance; n = number of fruit fly specimens of the reference species attracted in a given treatment; and N = total number of flies captured in the same treatment of the reference species.

#### 2.4.2. Estimation of Population Density of Fruit Fly Species

Species data collected from each treatment was reported using the variable “fruit flies per trap per day” (FTD) (Formula (2)), which describes the adult population size of the attracted fruit fly species at the sampling sites [19,27] given by:

(2) $$\mathrm{FTD}\;=\;\frac{F}{T\;\times \;D}$$

where F = number of fruit flies captured in the trap used for the treatment; T = number of traps used for the treatment; and D = average number of days of exposure of traps in the field.

#### 2.4.3. Determination of Sex Ratios

For each treatment, the total number of males and females of each species was recorded, and the sex ratio was expressed as the percentage proportion of males to females. This procedure was done for each fruit fly species in each treatment independently [31].

#### 2.4.4. Estimation of the Infestation Indices of Guava Fruit

The infestation indices were estimated by calculating the number of pupae and adults per kg of fruit [32,33] using the following formulae:

(3) $$\mathrm{Infestation}\;\mathrm{index}\;1\;(\mathrm{pupae}/\mathrm{kg})=\frac{total\;number\;of\;pupae\;harvested}{total\;kilograms\;of\;incubated\;fruits}$$

(4) $$\mathrm{Infestation}\;\mathrm{index}\;2\;(\mathrm{adults}/\mathrm{kg})=\frac{total\;number\;ofadults\;emerged}{total\;kilogramsr\;of\;incubated\;fruits}$$

The infestation indices of the different fruit sources were calculated independently and compared to each other.

#### 2.4.5. Correlation Between Trapped Fruit Flies and Adults/kg of Fruit

The FTD data were paired with the corresponding data on adults/kg of fruit, and Pearson’s correlation coefficient was calculated to assess the relationship between the two datasets. The correlation was made between the mean weekly FTD of the most abundant fruit fly species attracted in torula traps and the mean weekly fruit fly per kg of the same fruit fly species that emerged from the incubated guava fruits. When estimating this parameter, the overall infestation index (adults/kg) from fruits collected from the trees and from the ground was considered. The correlation analysis helped to evaluate whether trap catches could serve as reliable indicators of fruit infestation levels in the field, taking into consideration that food baits attract fruit flies from short distances. A regression analysis was later conducted to examine the relationship between the mean weekly FTD (independent variable) and the number of flies per kilogram of fruits (dependent variable).

### 2.5. Data Analysis

All statistical analyses were carried out using R software (R Core Team, 2025). The variable FTD was calculated for the first 12 weeks, when trap catches were high enough to detect statistical significance. On average, 5 flies per treatment were caught to make a total of 20 flies in all traps per week [34]. The data were subjected to analysis of variance (ANOVA) under a randomized complete block design, with treatment and block included as factors. When the assumptions of ANOVA (homogeneity of variances and normality of residuals) were not met, a logarithmic transformation [log (x + 0.5)] was applied to stabilize variances and improve normality [16]. Means were separated using Tukey’s honest significant difference (HSD) test at α = 0.05.

Detection sensitivity at low population densities was assessed using data from weeks 16–25 of the study period, when capture rates were low. For each trap observation, both total fruit fly capture (all species combined) and *Bactrocera dorsalis* counts were converted into binary detection variables, with values greater than zero coded as detection (1) and zero captures as no detection (0). Detection frequencies were then calculated for each treatment for both response variables. The differences in detection sensitivity among treatments were assessed using a chi-square test of independence, and Fisher’s exact test was used to confirm the robustness of the results. Post hoc pairwise comparisons were then conducted using Fisher’s exact test with Bonferroni adjustment.

The sex proportion of *Bactrocera dorsalis* was analyzed using a binomial generalized linear model (GLM) with a logit link, where female and male counts were specified as cbind (female, male). The model included treatment and week to assess the differences among treatments while accounting for temporal variation. The significance of treatment and week effects was evaluated using analysis of deviance (chi-square test). Additionally, separate models were fitted for each treatment to test whether the overall female proportion differed from 50% and to evaluate whether sex proportions varied over time within each treatment. The sex ratio of other species was not subjected to inferential statistical analysis due to the consistently low sample sizes throughout the study period, which reduced statistical power and increased uncertainty in the estimates, making formal hypothesis testing unreliable. Consequently, sex ratios for these species were described using observed proportions (percentages).

To assess the relationship between trap captures (FTD) and infestation levels (adults per kg of incubated fruit), the Pearson correlation coefficient was calculated. Correlation analyses were performed using the cor() function, and statistical significance was assessed with the cor.test() function, which provides a *p*-value and a 95% confidence interval for the correlation estimate [35]. A regression analysis was also performed to examine the relationship between mean weekly fruit flies per tap per day (FTD) as the independent variable and the number of emerged flies per kg of fruit as the dependent variable. Model assumptions, including normality of residuals and homoscedasticity, were checked prior to the interpretation of results.

## 3. Results

### 3.1. Absolute and Relative Abundance of the Different Fruit Fly Species

Throughout the study period (25 weeks), a total of 2844 fruit flies were attracted to the various treatments, from which 1670 were females and 1174 were males. The torula treatment attracted the highest number of fruit flies (1563) as the positive control, followed by palm sap (978) and molasses (299) while water was the least attractive (negative control). Three genera of fruit flies were identified, namely *Bactrocera* (2570 flies), *Ceratitis* (37 flies), and *Dacus* (237 flies). Within the genus *Bactrocera*, *Bactrocera dorsalis* was identified as the only and most abundant species, with a total of 2570 individuals, corresponding to a relative abundance of 90.37%. Within the genus *Dacus*, five species were identified, namely *Dacus bivittatus* (136, 4.78%), *Dacus frontalis* (53, 1.86%), *Dacus punctatifrons* (18, 0.63%), *Dacus vertebratus* (28, 0.98%), and *Dacus ciliatus* (2, 0.070%). Within *Ceratitis* the following species were identified: *Ceratitis quilicii (16*, *0.56%), Ceratitis rosa (10*, *0.35%)*, *Ceratitis capitata* (8, 0.28%), *Ceratitis punctata* (2, 0.07%), and *Ceratitis cosyra* (1, 0.04%).

Across treatments, *Bactrocera dorsalis* remained the dominant species, accounting for 92.33% in palm sap, 90.40% in torula, 84.28% in molasses, and 50.00% in water. The remaining species contributed only a small proportion of the total catches and were inconsistently distributed across treatments. Most of the minor species were more frequently recorded in the torula and palm sap traps.

### 3.2. Population Density of Fruit Fly Species in Each Treatment

The population density of the dominant species, *B. dorsalis,* differed significantly among bait treatments over the entire study period. Treatment had a highly significant effect on FTD (F_3,185_ = 53.96, *p* < 0.001), and a significant block effect was also detected (F_3,185_ = 10.48, *p* < 0.001), indicating variability among blocks.

The first positive control, torula yeast, had the highest FTD (4.15 ± 0.80) of *B. dorsalis*, confirming its position as a good attractant. The second was palm sap with a mean FTD of 2.61 ± 0.49. Molasses was third, with an FTD of 0.73 ± 0.12. Water, the negative control, was absolutely ineffective (0.01 ± 0.00 FTD).

**Figure 1:**
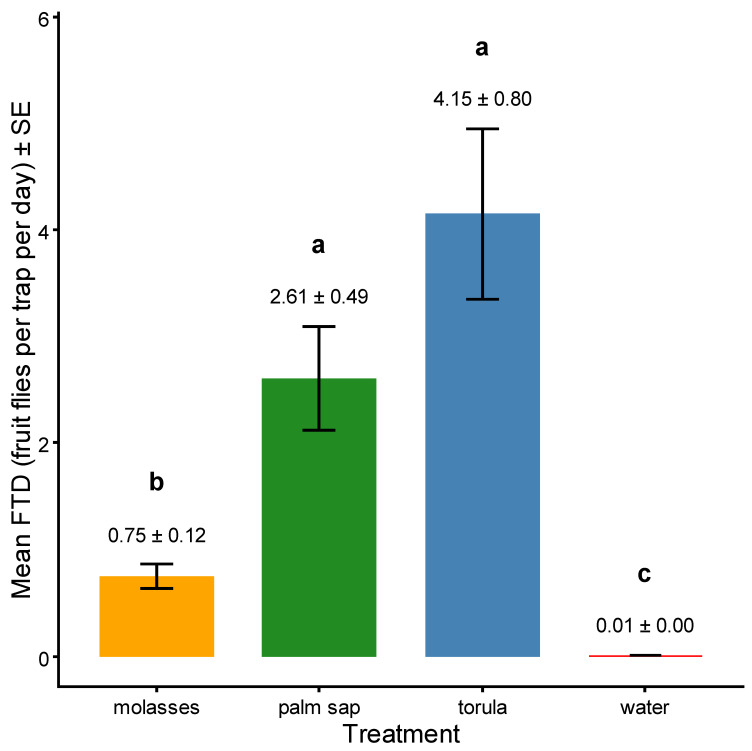
Mean FTD (fruit flies per trap per day) of *Bactrocera dorsalis* for the sampling period in each treatment. Treatments with bars with the same letter are not statistically different from each other, separated by Tukey’s test (*p* < 0.05). Error bars (whiskers) represent standard error (SE) of the mean.

**Figure 2:**
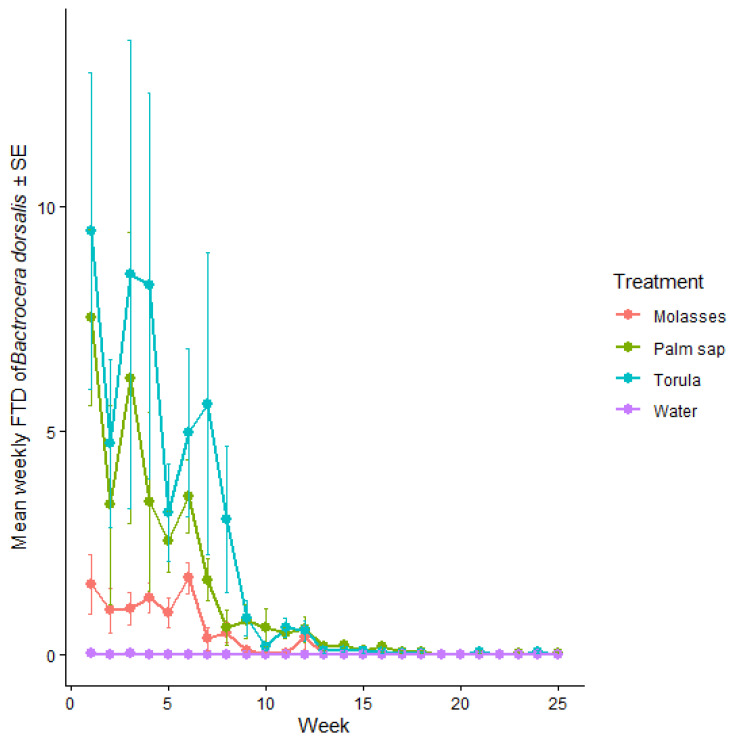
Mean weekly FTD (fruit fly per trap per day) of *Bactrocera dorsalis* per treatment with time (weeks). Error bars (whiskers) represent standard error (SE) of the mean.

Post hoc Tukey’s test for all treatment (baits) revealed that palm sap and torula did not differ significantly from each other, indicating that palm sap was as effective as the commercial bait in attracting *B. dorsalis*. Molasses displayed significantly lower attractiveness than palm sap and torula. Water was not attractive at all, as expected (Figure 1). The performance of the various attractants was consistent along every week of the study until there were almost zero catches of flies in the traps at about week 15 (Figure 2).

It can be seen from the graph that torula was consistently the best attractant followed by palm sap, molasses, and lastly water. It was also noted that for each treatment the mean weekly catches were the highest in the first week of the study and then decreased as the study period in weeks increased.

Significant differences in detection sensitivity were observed among treatments when all fruit fly species were considered (χ^2^ = 20.08, df = 2, *p* < 0.001; Fisher’s exact test, *p* < 0.001). Palm sap had the highest detection rate (57.5%), followed by torula (40%), while molasses showed limited sensitivity (10%). Post hoc pairwise comparisons indicated that both palm sap and torula had significantly higher detection sensitivity than molasses (adjusted Fisher’s exact test: molasses vs. palm sap, *p* < 0.001; molasses vs. torula, *p* < 0.05), whereas no significant difference was observed between palm sap and torula (*p* = 0.54). Similarly, for *Bactrocera dorsalis*, detection sensitivity also differed significantly among treatments (χ^2^ = 8.24, df = 2, *p* < 0.05; Fisher’s exact test, *p* < 0.05), although overall detection rates were lower. Palm sap remained the most effective treatment (25%), followed by torula (17.5%), while molasses showed very low detection (2.5%). Post hoc test showed that palm sap had significantly higher detection sensitivity than molasses (*p* < 0.05), while no significant differences were observed between palm sap and torula (*p* = 1.00) or between torula and molasses (*p* = 0.34). Accordingly, palm sap ranked the highest, torula showed intermediate performance, and molasses had the lowest detection sensitivity.

### 3.3. Sex Ratios of Fruit Flies

**Figure 3:**
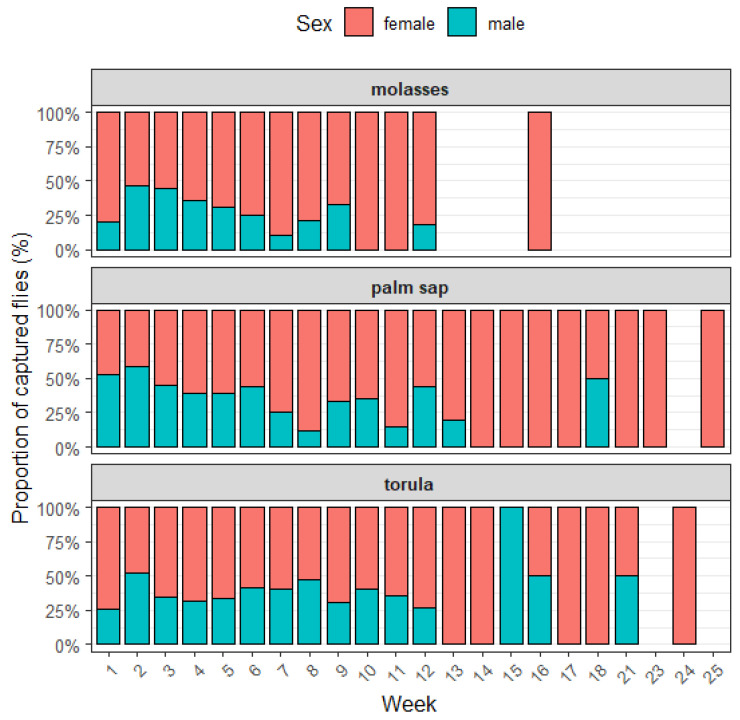
Weekly sex proportions of *Bactrocera dorsalis* captured under different treatments over the study period. Each column represents one week and is expressed as 100%, with stacked segments showing the relative proportions of females and males for each treatment.

Sex proportions of *B. dorsalis* differed significantly among treatments (χ^2^ = 24.60, *p* < 0.001) and across weeks (χ^2^ = 59.30, *p* < 0.001). Females were more abundant than males in all treatments, with estimated proportions of 70.24% in molasses, 56.48% in palm sap, and 63.98% in torula, all significantly different from 50% (molasses, *p* < 0.001; palm sap, *p* < 0.001; torula, *p* < 0.001). Across the study period, female proportion did not vary significantly in molasses (*p* = 0.229) but showed significant temporal variation in palm sap (*p* < 0.001) and torula (*p* < 0.001). The temporal trends in sex proportion for each treatment are illustrated in Figure 3, which shows the weekly variation in sex percentages across the study period.

In general, more females of the genera of *Bactrocera* and *Ceratitis* were attracted to the different treatments whereas more males of the genus *Dacus* showed a preference for them. Water captured only male flies (Table 1).

### 3.4. Infestation Indices of the Fruits

A total of 12,867 pupae were obtained from the fruits, 6154 from fruits collected from the ground, and 6713 from the trees. From the pupae a total of 10,928 adult fruit flies emerged, from which 5607 were females and 5321 were males. The infestation indices were calculated and recorded (Table 2).

Two genera of fruit flies were recovered from the incubated fruits. These were *Bactrocera* and *Ceratitis*, with *Bactrocera dorsalis* being the only species of the genus and the most abundant of all the emerged flies, while *Ceratitis* had three species. Flies of *Bactrocera dorsalis* accounted for 10,914 flies (99.87%), while the remaining 14 individuals (0.13%) comprised *Ceratitis capitata* (2), *Ceratitis quilicii* (8), and *Ceratitis rosa* (4).

### 3.5. Correlation Between Trapped Flies (B. dorsalis) and B. dorsalis Infestation Index

There was a statistically significant positive correlation between adult flies caught in traps and the adult flies emerged from fruits per kg (r = 0.652, t = 2.58, df = 9, *p* = 0.0297). The correlation is moderately high and indicates that a rise in the trap captures is associated with an increase in *B. dorsalis* infestation level. The 95% confidence interval for the correlation coefficient (0.0856–0.8999) also confirmed that the relation was consistently positive but with differential strength of association. These findings provide empirical evidence supporting the use of trap catches as a valid proxy for estimating infestation levels in the field, justifying their application in monitoring fruit fly population dynamics in orchard environments.

The regression model of a simple linear regression analysis was found to be statistically significant (F_(1,9)_ = 6.66, *p* = 0.0297), indicating that the population density of fruit flies in the field, as shown by mean weekly FTD, significantly predicts the number of emerged flies per kg of fruit.

The regression equation derived from the analysis is as follows:

Emerged flies per kg = 110.67 + 20.78 × mean weekly FTD.

**Figure 4:**
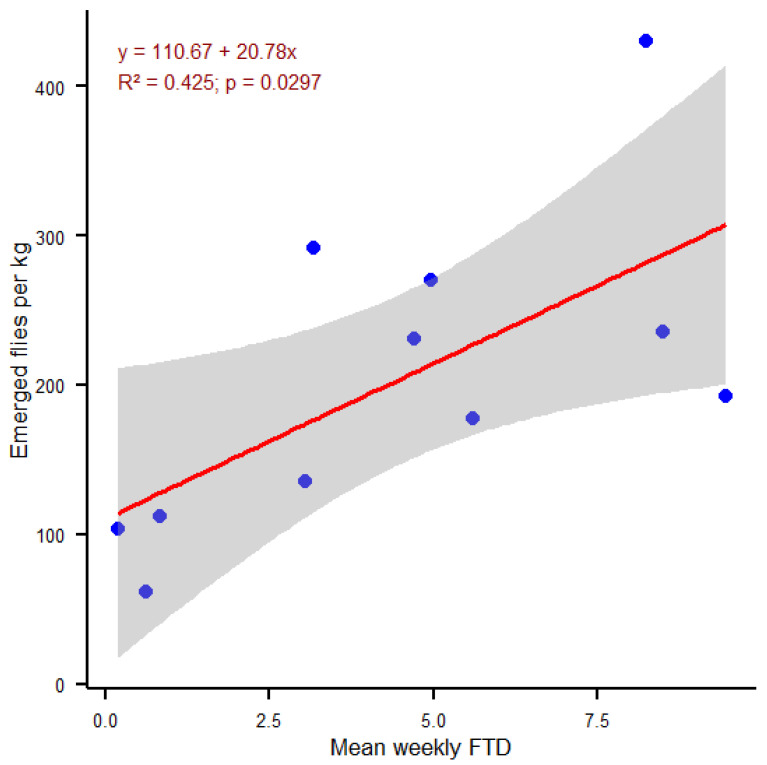
Regression of emerged flies/kg of *Bactrocera dorsalis* on mean weekly FTD (fruit flies per trap per day) of the same species of fruit flies attracted in torula treatment. The red line indicates the fitted linear regression model, while the grey shaded area represents the 95% confidence interval around the regression line.

This equation suggests that for every one-unit increase in the mean weekly FTD, the number of emerged flies per kg increases by approximately 20.78. The model explains 42.5% of the variation in the number of emerged flies per kg. (R^2^ = 0.425, adjusted R^2^ = 0.361), indicating a moderate positive association between the two variables. This is represented in Figure 4.

## 4. Discussion

*Bactrocera dorsalis* was identified as the most abundant species trapped in all food baits used. This is consistent with many studies, which reported dominance of the invasive *B. dorsalis* over the native fruit fly species in Africa ever since its invasion into Africa [12,36,37]. This dominance may be attributed to its high reproductive capacity, wide host range, and strong competitive ability over native species [12,38].

The findings also showed distinctly characterized sex-specific trends of fruit fly catches to the baits. For the *Bactrocera* and *Ceratitis* species, higher numbers of fruit fly females over males were captured. This is in agreement with earlier research in which females of the two genera had greater nutritional requirements, specifically protein and carbohydrate, in support of oogenesis and prolonged flight [39,40,41]. Fermentation-based attractants such as palm sap and torula yeast release compounds that stimulate feeding behavior, which may explain the higher capture of females. In contrast, the reverse was true for the species of *Dacus*, where more males were caught than females using the same food baits. The pattern is consistent with the results from [42], which showed that food baits, including torula, attracted more males of *Zeugodacus cucurbitae* flies than females. However, the results disagree with other studies in which more females were attracted than males [43,44]. This could be because there might have been more male flies in the field than females. Also, it is known that guavas are not good hosts for fruit flies of the genus *Dacus*, and so fewer females could be expected in the field because of fewer breeding sites [45]. However, flies of the genera *Dacus* and *Ceratitis* were not included in the statistical analysis of sex ratios due to the low number of individuals captured. According to the results, palm sap was found to be as effective as the commercial food bait torula, and thus represents a low-cost, locally available alternative, particularly in resource-limited sites. These results are consistent with those reported in ref. [46], which demonstrated that palm sap can serve as a low-cost alternative for fruit fly management in Ghana. The high attractiveness of palm sap to fruit flies in this experiment could be due to its natural chemical composition and fast fermentation rates. Fresh palm sap, when collected directly from the tree, is rich in soluble sugars, mainly sucrose, glucose, fructose, and proteins [47], which provide an immediately available source of energy for tephritid fruit flies. Such sugar-rich substrates play a crucial role in sustaining the flight activity, longevity, and reproductive potential of adult fruit flies [41]. Previous studies have demonstrated that carbohydrate and protein-rich food sources significantly enhance mating success, fecundity, and survival in tephritid species, underscoring their importance as potent feeding stimulants [41,44]. Apart from the composition of sugar, palm sap quickly becomes fermented by microorganisms during harvest, which is largely due to naturally occurring yeasts. This process results in the release of volatile metabolites like ethanol, acetic acid, and other esters, which act as chemical signals mimicking ripening or fermenting fruit and play a crucial role in host location by fruit flies. Furthermore, palm sap includes essential amino acids, vitamins, and minerals that facilitate active microbial growth and sustained production of volatile compounds [48]. Such nutritional adequacy guarantees that fermentation continues, keeping the bait’s appeal during extended trapping intervals, hence increasing its effectiveness. However, fermentation-based attractants are known to capture a wide range of non-target insects, which may affect trap efficiency and ecological interactions [49]. Although non-target insects were occasionally observed during trap inspections in this study, they were not quantified. Also, the performance of fermentation-based attractants may vary depending on preparation, fermentation stage, and environmental conditions, which can influence trap efficiency.

Detection sensitivity at low population densities is a key criterion for evaluating attractants used in monitoring. Our results demonstrate that palm sap exhibits higher sensitivity for *B. dorsalis* under low-density conditions, supporting its suitability for early detection and timely management interventions. In contrast, the low detection capacity of molasses suggests reduced reliability for monitoring at low population levels. This highlights the importance of selecting baits that remain effective when populations are low, as poor detection can lead to delayed management responses. Overall, palm sap shows potential as a sensitive and practical option for early warning in fruit fly monitoring systems, particularly *B dorsalis*, which is important for timely decision-making in integrated pest management systems.

For the infestation indices, the results indicate that fruits collected from the ground had a statistically lower number of adults per kg compared to those collected from the trees, while the pupae/kg was not statistically different. Earlier research has shown that fallen fruits tend to degrade faster and to become more susceptible to fungal and bacterial infections, and they are more exposed to predators, which collectively lowers the chances of larvae surviving, as well as the quality of pupae [19,50]. Also, during the harvesting of pupae from incubated fruits, many coleopterans and other organisms were observed in containers holding fruits collected from the ground. This may also have affected the quality of pupae and their emergence rate. Predation by soil-inhabiting coleopterans has been observed in studies of ground-exposed pupae of *Drosophila suzukii* and other fruit flies, where ants, ground beetles, field crickets, etc., significantly reduced pupal survival in exposed fallen fruit [51]. This might explain why, even when the infestation index of pupae/kg was similar, the adults/kg was lower in fruits collected from the ground. On the other hand, fruits collected from the tree may have provided a more suitable environment, offering higher moisture levels and pulp quality, thereby supporting greater larval survival, good pupal quality, and higher emergence rates [52,53]. However, some studies suggest that fallen fruits can sometimes lead to similar or even higher emergence rates, especially if decomposition makes the fruit softer and easier for larvae to feed on [54,55]. The overall mean infestation indices (245.1 ± 16.1 pupae per kg; 208.5 ± 13.3 adults per kg) suggest a high level of infestation in the collected fruits and confirm that guava is a preferred host for *B. dorsalis* [7]. Surveys based on incubation show varying results depending on the host plant and location. From a management point of view, these findings emphasize the importance of keeping orchards clean. Fallen fruits act as reservoirs of the developing stages of fruit flies and act as a source of infestation if left unattended [56,57]. As a result, combining sanitation practices with other strategies like using protein baits and introducing natural predators can lead to a more effective way of controlling fruit fly populations. These recommendations for management are consistent with integrated fruit fly control strategies used in African agricultural ecosystems, where high infestation levels and high emergence from incubated fruits have been previously documented [19,58].

The moderate but statistically significant positive correlation between the number of fruit flies captured in traps and the number of adult flies emerging per kilogram of fruit suggests that trap data may provide a useful indication of fruit fly activity. This finding aligns with previous studies. For example, Vayssières et al. [59] and Chen et al. [60] observed that higher trap catches of *fruit flies* were associated with higher fruit infestation in mango and guava orchards, respectively, and demonstrated that trap counts can be used to predict infestation risk. In ref. [59], trap captures were positively correlated with fruit infestation levels in mango (r = 0.68), showing that trap density reflected field infestation trends. Similarly, in ref. [60], female trap catches of *Bactrocera dorsalis* were positively correlated with guava fruit infestation (r = 0.322), and logistic regression indicated that each increase in female density class raised the odds of infestation by 34.5%, supporting the use of trap data for predicting infestation risk. Also, Manrakhan et al. reported that in citrus orchards, food-based traps captured females and more accurately reflected actual fruit infestation, particularly during fruit ripening [61]. The guidelines in ref. [27] also emphasize that trap indices, such as flies per trap per day, are widely used as proxies for population pressure in integrated pest management programs, although they do not fully capture all aspects of infestation. This was also demonstrated by the results of the present study, as the determination coefficient indicates that 42.5% of the variation in the number of flies emerging per kg of fruit is explained by the mean weekly FTD, suggesting that the model has moderate predictive ability. These results agree with earlier research [60], in which higher trap catches were linked to higher fruit infestation. The moderate coefficient of determination indicates that a substantial proportion of variation remains unexplained, likely due to factors such as fruit availability, environmental conditions, and surrounding host plants like mangoes, tropical almond, and bananas, which were within the vicinity of the orchard. These hosts may serve as alternative feeding or breeding sites and may contribute to the movement of adult flies into the study orchard. Therefore, the relationship observed between trap catches and infestation levels should be interpreted within a broader landscape context, rather than as a result of orchard conditions alone. Overall, mean weekly FTD provides useful information on population trends and infestation indices but should be used alongside other indicators when making pest management decisions. Additionally, it is clear that area-wide management programs, rather than localized ones, are more likely to provide effective control of fruit flies.

## 5. Conclusions

This study concluded that *Bactrocera dorsalis* was the most abundant species, consistent with its status as an invasive species. Generally, more females of *Bactrocera dorsalis* were captured through different treatments. Palm sap is as effective as torula yeast in attracting fruit flies, confirming its potential as a low-cost alternative for resource-limited farmers. Its detection sensitivity at low population densities was the highest among the tested baits. In addition, higher mean weekly FTD corresponds to higher numbers of flies emerging per kg of guavas.

## Figures and Tables

**Table 1:** Percentage of female (F) and male (M) fruit flies caught in each treatment.

Species	Treatment							
	Molasses		Palm Sap		Torula		Water	
	F (%)	M (%)	F (%)	M (%)	F (%)	M (%)	F (%)	M (%)
*Dacus bivittatus*	0.00	100	33.36	63.64	31.63	68.37	0.00	0.00
*Dacus punctatifrons*	0.00	100	12.50	87.50	25.00	75.00	0.00	100
*Dacus frontalis*	17.65	82.35	26.92	73.08	44.44	55.56	0.00	100
*Dacus vertebratus*	0.00	100	20.83	79.17	50.00	50.00	0.00	0.00
*Dacus ciliatus*	0.00	0.00	100	0.00	100	0.00	0.00	0.00
*Ceratitis quilicii*	0.00	0.00	100	0.00	93.33	6.67	0.00	0.00
*Ceratitis rosa*	0.00	0.00	50.00	50.00	100	0.00	0.00	0.00
*Ceratitis capitata*	0.00	0.00	100	0.00	100	0.00	0.00	0.00
*Ceratitis punctata*	0.00	0.00	100	0.00	100	0.00	0.00	0.00
*Ceratitis cosyra*	0.00	0.00	0.00	0.00	0.00	100	0.00	0.00

**Table 2:** Number of adults, females, and males emerged from the fruits and adult infestation indices of fruit flies in guavas. Means which share the same letter in the same column are not statistically different from each other, while means in the same column with different letters are statistically different from each other.

Fruit Source	Total Number of Fruits	Total Weight (kg)	Number of Pupae	Emerged Adults	*Bactrocera dorsalis*	Females	Males	Pupae/kg ± SE	Adults/kg ± SE
Guava (ground)	456	29.568	6154	5120	5115	2719	2401	210.0 ± 16.6 a	175.0 ± 13.7 b
Guava (from tree)	364	22.013	6713	5808	5799	2888	2920	287.0 ± 28.3 a	248.0 ± 23.1 a
Welch two-sample *t*-test								*p*-value 0.08346	*p*-value 0.03775
Total (overall)	820	51.581	12,867	10,928	10,914	5607	5321	245.1 ± 16.1	208.46 ± 13.3

## Data Availability

The raw data supporting the conclusions of this article will be made available by the authors on request.

## Abbreviations

The following abbreviations are used in this manuscript:

CABI	Center for Agriculture and Bioscience International
FAEF	Faculty of Agronomy and Forestry Engineering
FAO	Food and Agriculture Organization
FTD	fruit fly per trap per day
IAEA	International Atomic Energy Agency
